# Estimating aspect ratio of the distal femur and proximal tibia in the Emirati population from MRI scans of the knee: a preliminary experience

**DOI:** 10.1038/s41598-023-31715-4

**Published:** 2023-03-18

**Authors:** Ivan James Prithishkumar, Taiceer Abdulwahab, Alshafi Mohammad, Ali Albelooshi

**Affiliations:** 1 College of Medicine, Mohammed Bin Rashid University of Medicine and Health Sciences, Dubai, UAE; 2 Mediclinic City Hospital, Dubai Healthcare City, Dubai, UAE

**Keywords:** Anatomy, Musculoskeletal system

## Abstract

Size and shape of knee implants play an important role in the success of total knee arthroplasty. Several studies have identified anthropometric differences of the distal femur and proximal tibia between the genders and ethnicities. Ill-fitting prosthesis can cause overhang or under-fit resulting in persistence of pain, periprosthetic fracture and decreased range of motion. The purpose of this study was to estimate the aspect ratio of distal femur and proximal tibia in the Emirati population and determine whether gender differences exist within this group. Magnetic resonance imaging datasets of unilateral knees scans performed on adult Emirati patients at a tertiary care hospital were retrospectively examined. Knee parameters were obtained from 65 males and 46 females (n = 111). Females showed significantly smaller AP and ML dimensions of distal femur and lower aspect ratios compared to males (p < 0.001). Proximal tibial dimensions (AP and ML) of Emirati women are also significantly smaller compared to men. However, aspect ratio of proximal tibia did not show gender variation (p = 0.956) within the Emirati population. Emirati knees showed significant gender differences in bony dimensions and aspect ratio of the knee, and also have smaller aspect ratios when compared with most other population groups.

## Introduction

Total knee arthroplasty (TKA) is a very complex procedure done to alleviate pain and improve knee joint function in patients with osteoarthritis^1,2^. Success of TKA depends to a large extent on prostheses selection, accurate sizing and proper placement of the components^3^. The most important factor that determines sizing of the knee implant is the aspect ratio of distal femur and proximal tibia. Several investigators have studied the anthropometry of knee using varied techniques (CT, MRI, cadaveric dissection and intraoperative) and in diverse ethnic groups such as the Caucasian, Thai, Chinese, Japanese, Indian, Brazilian, and Korean population^4–15^. Most of these studies report significant differences in the aspect ratio between the population groups and between the sexes. Several studies have shown that for a fixed anteroposterior length of distal femur, women have narrower mediolateral widths compared to men^13,14,16,17^. Anatomic differences of the knee between the genders has raised associated implications and the need for a gender specific knee prosthesis^14^. Many of the above studies also report component mismatch between the dimensions of knee and currently available knee arthroplasty systems. This has led many surgeons to believe that implants based on data from the Western population may not be suitable for use in other racial groups, and that alterations in implant design are required to suit gender and ethnic variations^6,7,18^.

There have been no studies reporting on the aspect ratio from the Emirati population though total knee replacement is an increasingly common procedure performed in the UAE especially among elderly women with osteoarthritis. Emirati patients typically present late with severe end-stage osteoarthritis. However, they continue to maintain a high degree of knee flexion with a lax posterior capsule due to social and religious practices. This laxity has been observed to be more pronounced in female patients, who form the majority of those requiring knee replacement surgery. One technique in such cases is to upsize the femur using the anterior reference, but this would result in implant overhang in the absence of a narrow design femur. Therefore, one is often forced to use a larger polyethylene insert, subsequently raising the joint line by resecting more distal femur. This can lead to patella baja and mid flexion instability^4^.

The main objective of this study was to determine the distal femoral and proximal tibial dimensions of the Emirati population using MRI and determine if significant gender differences exist within the local Emirati population. There is no available data from the Emirati population, and hence morphometric data from this population will help to design a better-fit prosthesis and prevent overhang or underfit of prosthetic components after knee replacement. The study also determined if a gender-specific implant is necessary for this population and compares the aspect ratio of distal femur and proximal tibia with other racial groups.

## Methods

The study was approved by the institutional research ethics committee (DHCR RERC Research Permit Reference No.: RP2-03–10,032,015) of Mediclinic Middle East. Waiver of informed consent and Health Insurance Portability and Accountability act (HIPAA) was also authorized. All experiments were performed in accordance with relevant guidelines and regulations and in accordance with the Declaration of Helsinki. Magnetic resonance imaging (MRI) datasets of adult unilateral knees scans performed at a tertiary care hospital sequentially between Jan 2018 and June 2018 were retrospectively examined. Adult patients with healthy knees, normal lower limb alignment, and without degenerative osteoarthritis were included in the study. Patients with any kind of rheumatism, gross congenital or acquired deformity, concomitant hip and spine deformities, lower limb trauma or severe osteoarthritis were excluded. All anthropometric measurements of the distal femur and proximal tibia from the MRI images, were obtained by a single investigator using the OsiriX-MD software (MacOS, version 2.9) and entered into an excel sheet. Measured variables were presented as mean, range and standard deviation. Student t-test was done to determine if gender differences exist in the femoral and tibial dimensions. P-value < 0.05 was considered as statistically significant. MAC SPSS Statistics v20 and R core team was used to conduct the statistical analysis.

### Distal femur measurements

**Figure 1:**
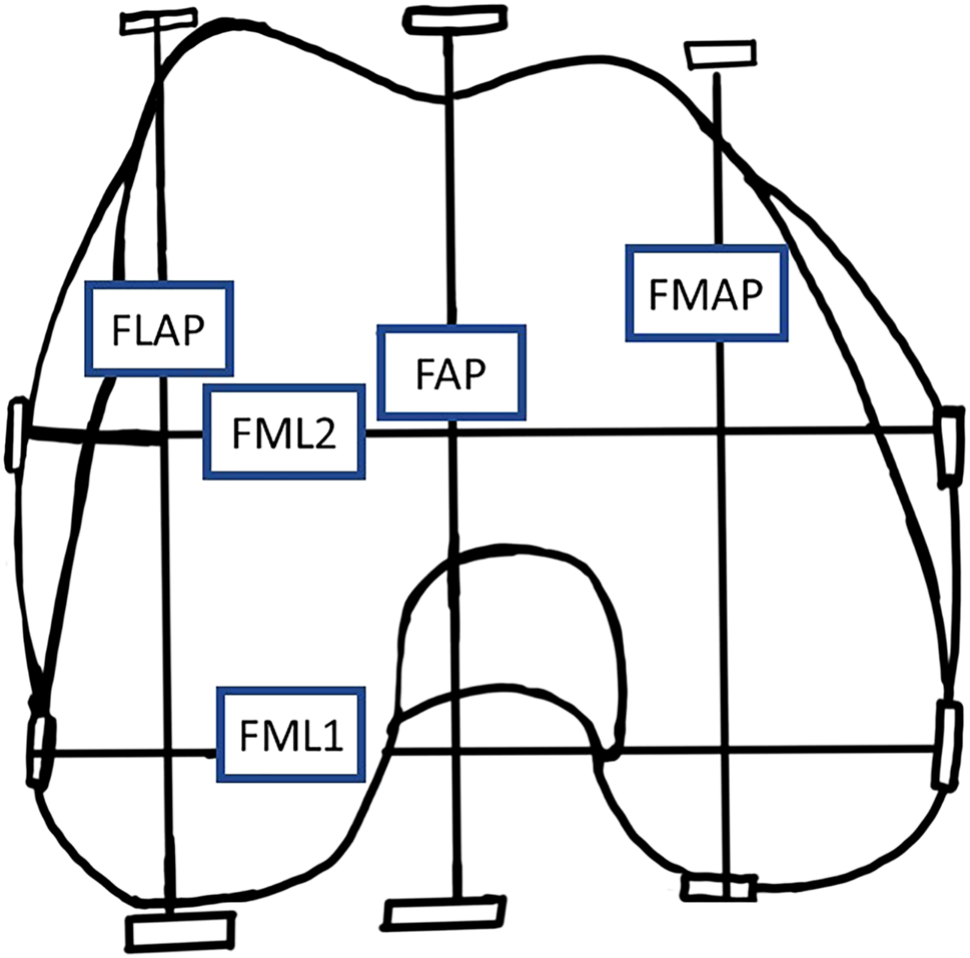
Measured dimensions of the distal femur. FAP—widest length of lateral femoral condyle; FML1—maximum distance between outer surfaces of medial and lateral condyle. FML2—distance between prominences of medial and lateral epicondyles; FLAP—widest anteroposterior length of lateral condyle; FMAP—widest anteroposterior length of medial condyle.

To simulate the distal femoral cut, an axial view taken approximately 9–10 mm above the lowest point of the medial femoral condyle was used for measurement. This is where the femoral component is inserted during total knee arthroplasty (TKA). The mediolateral dimension of distal femur was measured by two methods: FML1, as described by Chaichankul et al.^17^, is the maximum dimension between the outer aspects of medial and lateral femoral condyle; FML2, was measured as the longest length between the prominences of the medial and the lateral epicondyles. The antero-posterior dimensions of the medial femoral condyle (FMAP) and lateral femoral condyle (FLAP) were measured as the widest distance between the anterior and posterior edge of the respective condyles along an axis perpendicular to FML2 axis (Fig. 1). For all calculations of aspect ratio, FML1 was used as the femoral mediolateral width (FML) and FLAP was used as the maximum femoral anteroposterior length (FAP) of distal femur.

### Proximal tibia measurements

**Figure 2:**
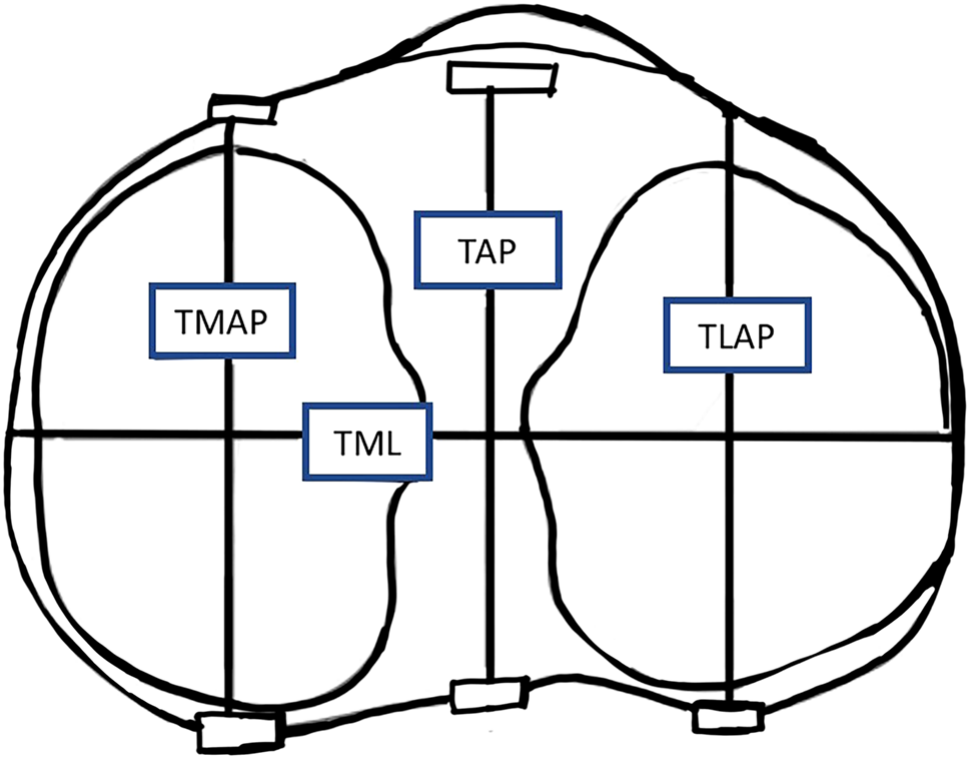
Shows the dimensions of proximal tibia. TML—widest mediolateral length; TAP—midline anteroposterior (AP) length perpendicular to TML axis; TLAP—widest AP length of lateral tibial condyle; TMAP—widest length of medial tibial condyle.

To simulate the distal tibial cut, an axial view 10 mm below the highest point of the lateral tibia plateau was used for measurement (Fig. 2). This is usually where the tibia component is inserted during a TKA. The mediolateral width of the proximal tibia was measured as the widest horizontal distance between medial and lateral margins of the tibial surface (TML). The anteroposterior dimensions of the tibial surface was measured as the maximum distance between the anterior and posterior margins of the proximal tibial surface perpendicular to the axis of TML^17,19^. The anteroposterior dimensions of tibia were measured at three locations, the medial tibial condyle (TMAP), lateral tibial condyle (TLAP), and in the midline (TAP).

### Aspect ratio

The aspect ratio of distal femur was defined as the ratio between the maximum mediolateral width (FML) and the maximum anteroposterior length (FAP) given by the formula FAR = FML/FLAP. Similarly, the aspect ratio of proximal tibia was defined as the ratio of tibial mediolateral width (TML) to the tibial anteroposterior length (TAP), given by the formula TAR = TML/TAP.

### Ethics declarations

All experiments were performed in accordance with relevant guidelines and regulations, abiding by the Declaration of Helsinki, and was in line with the regulations of the institutional research and ethical committees at Mediclinic Middle East.

## Results

Knee MRI scans of 111 Emirati patients were analysed within the study period. It included 65 men (58%) and 46 women (42%) with a male: female ratio of 1.4:1.0. The final sample closely represents the gender proportions found in the native Emirati population^20^. Mean age in men was 38 years (19–81 years) and in women was 50 years (18.5–85 years). Average height was 173.5 cm (161–189 cm) in men and 155.8 cm (140.5–174 cm) in women. Mean weight was 87.8 kg in men (59–133 kg) and 73.5 kg in women (56–92 kg). Mean BMI was 29.5 in men (19–44) and 30 in women (19–38). Table 1 illustrates the measured knee parameters in both genders.

**Table 1:** Compares measured parameters of the knee of both genders in the Emirati population.

	Total	Range	Males (Mean ± SD)	Range	Females (Mean ± SD)	Range	p-value
Femur							
Femoral ML	69.05 ± 6.06	57–81.4	73.05 ± 4.01	63.2–81.4	63.40 ± 3.32	57.0–69.6	p < 0.001
Femoral AP (FLAP)	60.88 ± 4.94	48.2–71.8	63.75 ± 3.70	56.0–71.8	56.83 ± 3.37	48.2–64.8	
Tibia							
Tibial ML	44.94 ± 4.27	34.7–56.1	77.22 ± 3.51	69.1–85.6	67.6 ± 3.53	60.4–77.5	p < 0.001
Tibial AP	42.54 ± 4.12	33.9–52.7	47.4 ± 3.10	41.3–56.1	41.5 ± 3.19	34.7–51.0	
Aspect ratio (AR)							
Femoral AR	1.13 ± 0.06	0.99–1.32	1.14 ± 0.05	1.01–1.32	1.11 ± 0.05	0.98–1.23	p = 0.001
Tibial AR	1.63 ± 0.09	1.43–1.90	1.63 ± 0.07	1.4–1.8	1.63 ± 0.09	1.4–1.9	p = 0.956

### Distal femur measurements

Measured values of distal femur are shown in Table 1. Mean femoral mediolateral (FML) measurements defined by FML1 was 73.05 ± 4.01 mm in men, and 63.40 ± 3.32 mm in women; FML2 was 82.6 ± 4.0 mm in men and 71.6 ± 3.1 mm in women The mean anteroposterior (FAP) value was 63.75 ± 3.70 mm in males and 56.83 ± 3.37 mm in females. The mean medial femoral condylar length (FMAP) and lateral femoral condylar (FLAP) were 56.91 ± 4.72 mm and 63.75 ± 3.70 mm respectively in men. The mean FMAP and FLAP lengths were 50.65 ± 3.91 mm and 56.83 ± 3.37 mm respectively in women. Overall, males had significantly larger femoral dimensions when compared with women (p < 0.001).

**Figure 3:**
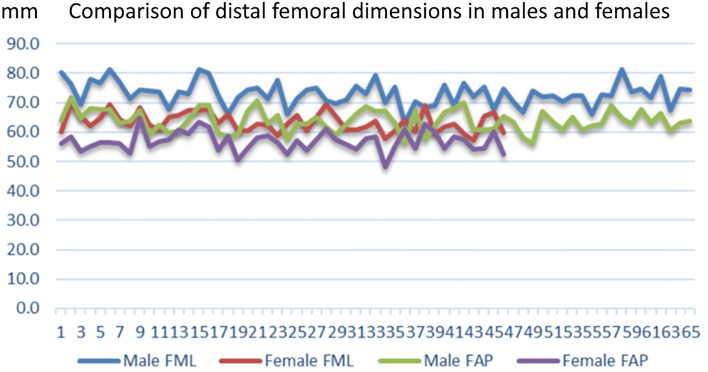
Graph shows gender variation in measured parameters of distal femur.

Figure 3 illustrates the gender-wise representation of distal femoral dimensions. For both mediolateral and antero-posterior dimensions, it is clearly evident that males have significantly larger dimensions (p > 0.001).

### Proximal tibia measurements

Measured variables of proximal tibia are shown in Table 1. The mean tibial mediolateral width (TML) and anteroposterior length (TAP) were 77.22 ± 3.51 mm and 47.40 ± 3.10 mm, respectively in men. The mean TML and TAP was 67.60 ± 3.53 mm and 41.50 ± 3.19 mm, respectively in women. The mean lateral condylar AP dimension (TLAP) and medial condylar AP dimension (TMAP) of proximal tibia was 45.90 ± 3.02 mm and 50.9 ± 3.69 mm respectively in men, and 39.20 ± 3.04 mm and 44.43 ± 2.8 mm respectively in women. There were significant gender differences in dimensions of proximal tibia. Females had significantly smaller values compared with men for every measured variable (p < 0.001).

### Aspect ratios

The mean femoral aspect ratio (FAR) was 1.14 ± 0.05 in men and 1.11 ± 0.05 in women with significant gender variation in aspect ratio of distal femur (p = 0.001). However, mean tibial aspect ratio (TAR) was 1.63 ± 0.07 in males and 1.63 ± 0.09 in females, statistical analysis indicating no significant difference between the sexes (p = 0.956).

**Figure 4:**
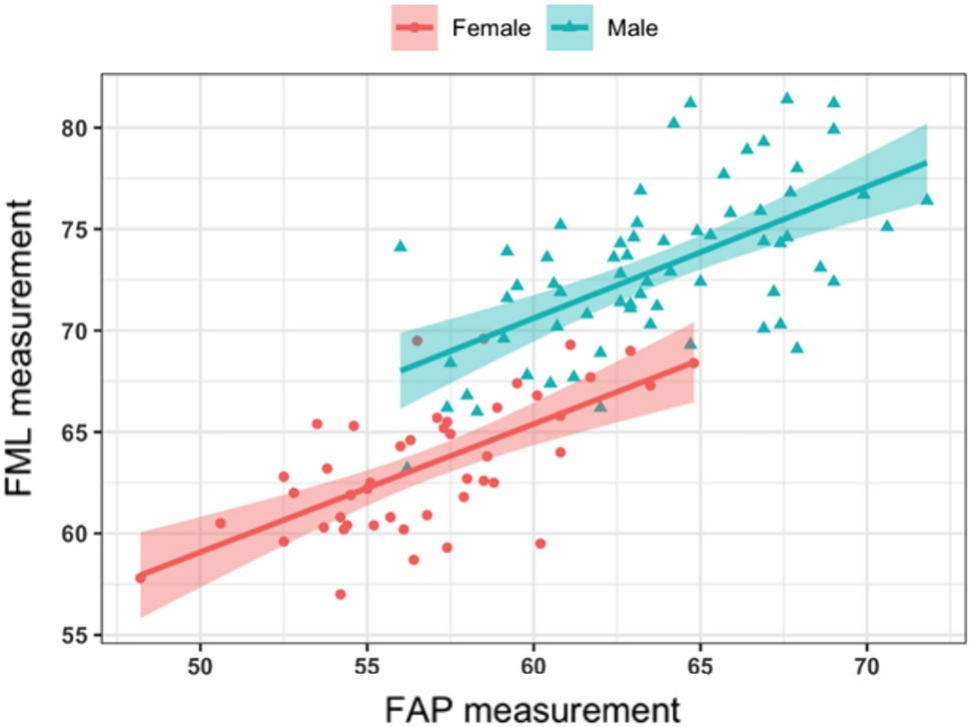
Shows distribution of femoral aspect ratio (FML/FAP) in males and females.

Figure 4 shows the scatter plot of distribution of aspect ratios in the male and female groups. It is clearly evident, that both AP and ML femoral dimensions are significantly larger in males compared to females, thus indicating that males have significantly larger sized knees than females. Linear regression analysis showed an r^2^ of 0.35 for males and 0.40 for females.

Table 2 illustrates the age-wise distribution of measured knee parameters in the whole group (age range: 18.5–85.8 years). Statistical analysis showed no significant difference in distal femur (p = 0.67) and proximal tibia (p = 0.350) dimensions between the age groups, indicating that an individual’s size of knee does not show much variation after attaining 18 years of age.

**Table 2:** Age-wise distribution of mean/SD of measured parameters and aspect ratio.

Age groups (years)	No	Femur ML	Femur AP	Femur aspect ratio	Tibia ML	Tibia AP	Tibia aspect ratio
10–19.9	4	66.4	60.5	1.09	71.9	43.3	1.67
20.0–29.9	27	71.3	62.4	1.14	75.5	46.5	1.62
30.0–39.9	29	70.2	61.9	1.13	74.6	45.4	1.64
40.0–49.9	15	67.1	60.3	1.11	71.9	44.4	1.61
50.0–59.9	12	68.7	59.8	1.14	70.8	44.7	1.58
60.0–69.9	13	66.0	58.4	1.13	70.9	43.2	1.64
70.0–79.9	9	68.7	59.7	1.15	72.4	43.6	1.66
80.0–89.9	2	65.5	57.5	1.14	68.2	43.0	1.58

## Discussion

Data emerging from this study shows that females have significantly smaller AP and ML dimensions of distal femur compared to males (p < 0.001). Mean femoral mediolateral (FML) measurements was 73.05 ± 4.01 mm in men, and only 63.40 ± 3.32 mm in women. The mean anteroposterior (FAP) value was 63.75 ± 3.70 mm in males and only 56.83 ± 3.37 mm in females. The mean medial and lateral condylar lengths were also significantly larger in men. Overall, males had significantly larger femoral dimensions compared with women (p < 0.001). The mean femoral aspect ratio (FAR) was 1.14 ± 0.05 in men and 1.11 ± 0.05 in women, again showing significant gender variation and larger ratios in Emirati men. Measurements of proximal tibia also clearly indicate that Emirati women have significantly smaller AP and ML dimensions compared to men (tAble 1). The mean tibial mediolateral width (TML) was 77.22 ± 3.51 mm in men and only 67.60 ± 3.53 mm in women; mean anteroposterior length (TAP) was 47.40 ± 3.10 mm in men and only 41.50 ± 3.19 mm, respectively in women (p < 0.001). However, the aspect ratio of proximal tibia did not show any gender variation (p = 0.956) within the Emirati population.

Total knee replacement is becoming a more commonly performed procedure within the UAE, particularly over the last decade. There are several reasons contributing to this, including an increasing elderly population with a higher life expectancy, increased accessibility and awareness of medical facilities and procedures, a larger group of younger, active and physically demanding patients who undergo total knee replacement to help alleviate end-stage symptoms enabling functional return of activities, and a general overall acceptance of knee arthroplasty as an excellent procedure to improve pain, function and mobility in end-stage arthritis^18,21^. Since most knee implants in the market are generally tailored for those of European descent, our objective was to estimate dimensions of the Emirati knee, compare morphological data with other ethnic populations, and investigate differences in knee morphometry between genders within the Emirati population^22^. Identification of differences are crucial to gender and ethnic specific design and longevity of implant.

We compared the results of our study with a meta-analysis done by Kim et al.^4^, among knees from different ethnic groups (Table 3). The meta-analysis shows that Caucasians have the widest ML dimensions and Indians the least. While comparing mean FAP values, those of African descent have the largest AP values followed by East Asians and Indians who have the smallest AP value. However, on comparing mean aspect ratios, we found that Emirati knees have significantly lower values compared to all other racial groups (Emirati 1.13 vs Caucasian 1.20). On comparing absolute values, Emirati FML dimensions are much lesser in both genders when compared to Caucasian, East Asian and Black communities, but wider than Indians (Emirati 69.0 vs Caucasian 74.0). Emirati FAP values are significantly smaller than Caucasian and Black communities but larger than others (Emirati 60.8 vs Black 63.0). These differences may be attributed to differences in body height, build and genetic pre-disposition between the races^23^.

**Table 3:** Comparing distal femoral dimensions of the Emirati population with a meta-analysis of other ethnic populations.

Ethnicity	Femoral AP (3650 knees; 13 studies) (Mean and 95% CI)			FML (1884 knees; 15 studies) (Mean and 95% CI)			Femoral aspect ratio (4825 knees; 14 studies) (Mean and 95% CI)		
	Male	Female	Whole group	Male	Female	Whole group	Male	Female	Whole group
White	64 (60–69)	59 (54–64)	62 (57–66)	79 (75–83)	69 (65–72)	74 (70–77)	1.22 (1.13–1.31)	1.17 (1.08–1.26)	1.20 (1.11–1.29)
Black	66 (61–70)	61 (55–67)	63 (58–68)	71 (65–77)	67 (60–75)	69 (64–74)	1.19 (1.09–1.29)	1.19 (1.08–1.26)	1.20 (1.11–1.29)
East Asian	61 (57–66)	56 (52–60)	59 (54–63)	76 (73–79)	67 (64–70)	71 (69–74)	1.27 (1.18–1.35)	1.23 (1.15–1.32)	1.25 (1.16–1.34)
Indian	61 (45–77)	55 (39–70)	59 (42–73)	70 (59–80)	61 (49–73)	65 (55–76)	–	–	–
Emirati (Mean/Range)	63.75 (56–71)	56.83 (48–64)	60.88 (48–71)	73.05 (63–81)	63.40 (57–69)	69.05 (57–81)	1.14 (1.0–1.32)	1.11 (0.98–1.23)	1.13 (1.0–1.32)

There are a few limitations to this study. Only 16% of the UAE population consist of native Emiratis—the others include persons from other Arab nations (18%), Asians (60%), and western expats (12%). We selected only Emiratis as eligible candidates for the study. In addition, our study included healthy non-pathological knees of persons from a wider age group and may present slight differences from the morphology of osteoarthritic knees affecting the elderly population. In conclusion, wide variations in the morphometry of knee occurs between racial groups and the genders. More studies need to be done that compares the knee parameters of the Emirati population and currently utilized knee arthroplasty systems in this population.

In conclusion, this preliminary study clearly reveals that the measured bony parameters of the Emirati knee are significantly smaller in the adult female compared with the adult male. The identification of gender differences is crucial and justifies the need to develop a gender specific design while manufacturing knee implants for the Emirati population. Wearing a better fit knee implant will result in significantly better clinical and functional outcomes and longevity of implant following a TKR.

## Data Availability

The de-identified datasets used and/or analysed during the current study are available with the corresponding author and can be made available on reasonable request.
